# The global COVID-19 mortality cost report card: 2020, 2021, and 2022

**DOI:** 10.1371/journal.pone.0284273

**Published:** 2023-05-11

**Authors:** W. Kip Viscusi

**Affiliations:** University Distinguished Professor of Law, Economics, and Management, Vanderbilt University, Nashville, TN, United States of America; US Department of Agriculture, UNITED STATES

## Abstract

The United States is the country with the greatest number of COVID-19 deaths in 2020, 2021, and 2022. Both the U.S. and the world exhibited an increase in the number of COVID-related deaths in 2021 and a decrease in 2022. The U.S. share of COVID-related deaths declined in 2021 but rose in 2022, leading to a cumulative total U.S. mortality share of 17%. The extent to which the U.S. is an outlier is even greater based on the monetized mortality costs. Using the value of a statistical life to monetize the mortality impact increases the performance gap between the U.S. and the rest of the world because of the high mortality risk valuation in the U.S. The worldwide COVID-19 mortality cost was $29.4 trillion as of January 1, 2023, with a U.S. share of $12.7 trillion, or 43% of the global total. Throughout the COVID pandemic, the U.S. mortality cost share has been in the narrow range of 43% to 45%. Given the high U.S. value of a statistical life, these monetized mortality cost values are more than double the U.S. share of COVID-related deaths. The U.S. mortality cost share is greater if the value of a statistical life declines more than proportionally with income for low-income countries.

## 1. Introduction

The COVID-19 pandemic generated an initial wave of illnesses and deaths. Countries throughout the world responded with a series of voluntary and mandatory precautionary measures, including lockdowns, masks, hybrid conferences, remote learning, social distancing, travel restrictions, limitations on activities, and vaccinations. Despite these precautionary policies, the adverse health impact of COVID-19 has been considerable. This article focuses on the pandemic’s mortality costs, which dwarf the price tag of the economic stimulus efforts launched in response to the pandemic. The United States has had a poor overall COVID mortality record. The relative U.S. performance in terms of total COVID-related mortality improved in 2021, but declined in 2022. This article monetizes the worldwide mortality costs from the COVID pandemic, for which the U.S. has had a 43% share.

## 2. Death rate statistics

Table 1 presents the number of deaths and mortality costs by year for the world and for the top 10 mortality costs countries in different time periods. The countries are ordered by their total monetized mortality costs in each period. The mortality cost valuations using the value of a statistical life (VSL) is the focus of Section 3. Panel A presents the number of deaths and mortality costs through 2020, Panel B presents the additional deaths and mortality costs in 2021, Panel C presents the cumulative totals for deaths and mortality costs through 2021, Panel D presents the number of deaths and mortality costs in 2022, and Panel E presents the cumulative totals for deaths and mortality costs through 2022. The two principal components of the mortality cost assessment are the number of deaths and the monetized value of each death by country.

**Table 1:** Mortality Impacts of COVID-19.

**Panel A** Mortality Impacts as of January 1, 2021		
**Country**	**Number of Deaths**	**VSL x Deaths (Millions $)**
World	1,828,556	8,979,510
United States	355,918	4,057,465
United Kingdom	74,125	654,977
France	64,765	534,683
Italy	74,621	498,585
Brazil	195,152	391,454
Germany	34,388	321,714
Spain	50,837	295,361
Mexico	125,807	248,769
Belgium	19,528	175,972
Canada	15,606	151,085
**Panel B** Change in Mortality Impacts in 2021		
**Country**	**Number of Deaths**	**VSL x Deaths (Millions $)**
World	3,598,075	13,113,608
United States	491,149	5,599,099
Brazil	423,957	850,413
Germany	78,390	733,371
United Kingdom	74,653	659,643
Russia	252,152	587,949
France	59,086	487,798
Italy	62,892	420,217
Mexico	173,621	343,316
Spain	38,568	224,079
Peru	165,010	205,988
**Panel C** Mortality Impacts as of January 1, 2022		
**Country**	**Number of Deaths**	**VSL x Deaths (Millions $)**
World	5,426,631	22,093,118
United States	847,067	9,656,564
United Kingdom	148,778	1,314,620
Brazil	619,109	1,241,868
Germany	112,778	1,055,085
France	123,851	1,022,481
Italy	137,513	918,802
Russia	309,707	722,152
Mexico	299,428	592,084
Spain	89,405	519,440
Argentina	117,169	297,305
**Panel D** Change in Mortality Impacts in 2022		
**Country**	**Number of Deaths**	**VSL x Deaths (Millions $)**
World	1,222,846	7,263,352
United States	270,921	3,088,499
Germany	48,687	455,487
United Kingdom	50,159	443,211
Italy	47,129	314,895
France	38,111	314,634
Japan	39,120	309,421
Russia	84,055	195,993
Australia	14,800	181,047
Canada	18,609	180,158
Spain	27,690	160,878
**Panel E** Mortality Impacts as of January 1, 2023		
**Country**	**Number of Deaths**	**VSL x Deaths (Millions $)**
World	6,649,477	29,356,470
United States	1,117,988	12,745,063
United Kingdom	198,937	1,757,831
Germany	161,465	1,510,572
Brazil	693,941	1,391,973
France	161,962	1,337,115
Italy	184,642	1,233,697
Russia	393,762	918,145
Spain	117,095	680,318
Mexico	331,099	654,710
Canada	48,948	473,877

The number of COVID-19 fatalities is drawn from the Worldometer [1] data, which is the principal dataset used for this analysis. This organization compiles data from official governmental sources throughout the world, such as the Ministry of Public Health Afghanistan and the Queensland Health Authority in Australia [2]. The COVID-19 fatality total pertains to the cumulative number of deaths among detected cases in each year. Criteria for whether a death is designated as a COVID-19 death vary by country and sometimes within country. For example, Manitoba, Canada offers the following guidance for being designated as a COVID-19 death, which is adapted from the World Health Organization (WHO) International Guidelines: “A death resulting from a clinically compatible illness, unless there is a clear alternative cause of death that cannot be related to COVID disease (e.g., trauma). There should be no period of complete recovery between illness and death.” Australia offers a simpler definition of a COVID-19 death: “A death that has been certified by a doctor as having been caused by the new Coronavirus strain.” In addition to differences in the definition of which deaths are attributable to COVID-19, there may also be differences in countries’ medical services and their vigilance in identifying and reporting whether a particular death is COVID-related, potentially biasing the reported pandemic fatality values.

The mortality estimates based on the Worldometer data align with those based on the WHO COVID-19 fatality estimates [3]. Whereas Worldometer assembles the fatality data based on information drawn from country websites and ministries of health, the WHO compiles its statistics based on reports that the countries make to the WHO. The WHO and Worldometer estimates may vary by country, but for the overwhelming majority of countries there are no differences in the reported fatality numbers. The total global numbers of COVID-related deaths are very similar. For example, the cumulative number of COVID-related deaths reported as of January 1, 2022 based on the WHO data was 5,453 lower than the Worldometer estimates, where this difference is 0.1% of the total number of COVID deaths.

As shown in Panel A of Table 1, the total global number of deaths attributed to COVID-19 through the end of 2020 was 1,829,556 based on the Worldometer [1] data. The global number of deaths in 2021 was almost double this amount, or 3,598,075, producing a cumulative total number of deaths at the end of that year of 5,426,631. COVID fatality rates declined in 2022, as there were 1,222,846 global deaths in 2022, leading to a cumulative total number of deaths of 6,649,477 as of January 1, 2023. For each of each of these time periods, the United States had the highest number of annual deaths and the highest cumulative death toll, which reached 1,117,988 as of January 1, 2023.

Even excluding deaths in the U.S., the surge and subsequent decline of total COVID mortality is substantial. The ratio of the cumulative number of global deaths as of January 1, 2022 to the cumulative total the previous year was 3.11 after excluding the U.S. death toll. The subsequent abatement of the death rate in 2022 is apparent as the global ratio (excluding the U.S.) of the cumulative number of deaths on January 1, 2023 relative to January 1, 2022 was 1.21.

The U.S. share of the global COVID deaths has fluctuated during the pandemic. After starting at a 19% share of global deaths through 2020, the U.S. accounted for only 14% of the global deaths in 2021, which has been the peak year of the pandemic. This improved relative U.S. performance had a favorable effect on the increase in the global death toll and reduced the U.S. to a 16% share of the cumulative global number of deaths through 2021. However, as the pandemic waned worldwide in 2022, the U.S. share of deaths rose to 22%, boosting the cumulative U.S. share of total COVID as of January 1, 2023, deaths to 17%.

## 3. Monetizing mortality costs

### 3.1 The value-of-a-statistical life measure

The dominant role of the U.S. in contributing to the global mortality cost of COVID becomes more striking after coupling the country’s leading mortality total with a high monetized value of the risk death. To provide an assessment of the magnitude of the economic cost of COVID-related mortality, one must monetize the costs of these deaths in a manner that reflects each country’s valuation of the risk. A reduction in the risk of death does not have an infinite value. Societies have limited financial resources and have competing uses for these funds, leading governments to explicitly or implicitly place a finite value on reducing different types of mortality risks. After recognizing that it is not feasible to make an unbounded financial commitment to policies that promote individual health, the task is then to determine the best approach for making this assessment. How to value these deaths depends on the underlying conceptualization of what is being valued. If the focus is on compensating the family for the financial loss associated with the death, then the present value of the lost earnings and medical costs is usually the appropriate measure for wrongful death awards. However, applying that measure in this context does not incorporate the welfare loss to those who have lost their lives because of COVID. More generally, societies’ willingness to pay to reduce risks of death goes beyond accounting measures such as the value of the income that the deceased will no longer be able to earn.

The principal economic approach used to value mortality risks takes a prospective perspective with respect to the willingness to pay to reduce mortality risks. Thus, rather than asking what the value *ex post* would be of compensating survivors for the certainty of a particular death, the focus instead is on the *ex ante* value per expected death that is prevented in situations involving small mortality risk reductions. Consider the following example. Suppose that there is a group of 10,000 people, each of whom faces a 1/10,000 risk of death, so that there would be one expected death to their group. If each person is willing to pay $900 to eliminate that risk, then collectively members of the group are willing to pay $9 million to prevent one expected death to some member in their group. In this example, the VSL is $9 million. The VSL serves as a measure of the rate of tradeoff between money and risk for small changes in risk. The VSL overstates how much people would be willing to pay to reduce the probability of death from 1.0 to zero, and it understates how much people would need to be compensated for an increase in the probability of death from 0 to 1.0. The underlying thought experiment that is adopted here in valuing COVID mortality is to monetize COVID-19 deaths in the same manner as would be the case if the government could undertake a series of policies that would generate small reductions in risk for any given individual, but collectively would lead to the overall reduction in fatalities. This approach is consistent with that used to value mortality risk reduction provided by policy by government agencies throughout the world and in other analyses of the mortality costs of COVID-19 [4–7].

Following the practices generally adopted by government agencies, the calculations presented here apply the average U.S. VSL to monetize the mortality risk reductions in the U.S. and the average country-specific VSL counterparts to monetize deaths in other countries. As indicated below, the procedure for constructing country-specific VSL levels is to use the U.S. average VSL as the reference point and then to transfer this value to other countries by incorporating different assumptions about the influence of a country’s income level on that country’s average VSL. The focus on average VSL statistics does not imply that there is a country-specific VSL pertinent to all people in a particular country. Even within the U.S., the VSL is not a single figure applicable to everyone. Preferences with respect to risk and willingness to pay to avoid these risks vary. There are many potential sources of heterogeneity in the VSL, which is a value that varies across the population and across countries. Application of average VSL levels across a country’s population is appropriate when the risks are broadly distributed. If the risks are concentrated among those with a lower VSL, as is the case of hazards affecting those with very short remaining life expectancy, then the pertinent VSL and total mortality costs may be lower. For example, to the extent that one is applying an age-adjusted VSL using the counterpart values of a statistical life year, the mortality cost of COVID-19 would be reduced by about half [5]. However, losses from other health impacts may offset such an adjustment. Monetized morbidity costs may equal or exceed the value of the age-related reduction in monetized mortality costs [5, 8] so that the mortality cost calculations do not overstate the overall health impact even with an age adjustment. Other estimates of the morbidity cost associated with COVID based on quality-adjusted life years that are lost also indicate that the monetized value of the losses associated with morbidity impacts are substantial [9]. Another possible refinement is that if the policies that are envisioned would lead to very large changes in risk levels, the applicable VSL would be reduced [5, 7], mostly because substantial risk-reducing expenditures may deplete available funds used for risk reduction. Such influences might have come into play had the government pursued a single, very costly policy that would have prevented all COVID-related deaths. The approach adopted here is to use the average VSL across a country’s population rather than incorporating such considerations. Note that to the extent that these adjustments affect the valuation of mortality risks in all countries similarly, the relative role of the U.S. and the rate of change in the mortality costs associated with the pandemic are unaffected by adjustments for age and non-incremental risk reductions. However, the absolute level of costs is dependent on the absolute levels of the VSL.

### 3.2 Assigning mortality cost values

The specific values that I am using to monetize the mortality rates use as the starting point my average U.S. VSL number of $11.4 million. This estimate is the value in December 2021 dollars, (calculated using the U.S. Bureau of Labor Statistics 2022 CPI Inflation Calculator) [10], of the VSL estimates that have been derived from a meta-analysis of VSL studies using labor market data to assess the wage increase that workers require to incur fatality risks [11]. U.S. government agencies rely on this meta-analysis in their regulatory impact analyses. The studies included are restricted to those using occupational fatality risk measures from the Census of Fatal Occupational Injuries, which is a comprehensive census of all U.S. occupational fatalities. The $11.4 million estimate incorporates adjustments for any biases due to publication selection effects [12]. This figure is similar to the estimates used by the U.S. Department of Health and Human Services [13] of $9.3 million in 2014 USD (https://aspe.hhs.gov/reports/updating-vsl-estimates) and the U.S. Department of Transportation [14] of $9.4 million in 2014 USD (https://www.transportation.gov/office-policy/transportation-policy/revised-departmental-guidance-on-valuation-of-a-statistical-life-in-economic-analysis). As noted above, a VSL of $11.4 million does not pertain to the amount that people would be willing to pay to avert their death. Rather, it is the aggregate willingness to pay for the small mortality risk reductions across the population of the type one could expect from a publicly funded vaccination effort. The VSL values fatalities by a greater amount than is usually awarded in wrongful death cases, as it generally exceeds the present value of workers’ lifetime earnings by about an order of magnitude.

The use of this average VSL based on labor market decisions involving occupational risks does not imply that all individual decisions regarding vaccinations and protections in the COVID pandemic reflected a sensible balancing of the risks and the costs of risk reduction consistent with the tradeoff implied by the VSL. As Bruin de Bruine et al. [15] document, individual beliefs and protective behaviors were based on different types of information, the filtering of this information through politically oriented outlets, and the political preferences of the individual. Application of the VSL in policy decisions based on accurate assessment of the risks and costs produces decisions that would be consistent with the types of mortality reduction policies for which the VSL is often used.

To transfer the U.S. VSL statistics to construct country-specific estimates of the VSL for other nations, it is helpful to have an established empirical basis for the relationship of the VSL across countries. A substantial literature has shown that the VSL is positively related to a country’s income level. Consequently, to assess the country-specific mortality costs of the pandemic, it is feasible to transfer the U.S. VSL estimate to other countries based on differences across countries in the GNI per capita. The data utilized here are retrieved from Viscusi and Masterman [16], where they report data from “the World Bank’s GNI per capita numbers calculated using the Atlas method which is based on exchange rates and inflation rates.” Differences in attitudes toward risk, religion, health care systems, and social norms may all contribute to national variation in the VSL. However, a major determinant of national VSL differences stems from differences in income, which is a characteristic for which there is available data as well as an economic literature documenting the income-VSL relationship. How the VSL differs from that in the U.S. depends on income levels and the country’s income elasticity of the VSL. The income elasticity of the VSL is defined as the percentage change in the country’s VSL in response to a 1 percentage change in the country’s income. Here the focus is on percentage differences in the country’s GNI per capita compared to the U.S.

The relationship between income and the VSL may vary across countries so that it is useful to explore a variety of such relationships. The primary set of estimates presented here uses an estimated income elasticity of the VSL 1.0, whereby a 1 percentage lower income level in a country will lead it to have a 1 percent lower VSL. This estimate of the average international income elasticity of the VSL is based on both a meta-regression analysis of labor market estimates of the VSL [16] and a meta-regression analysis of stated preference estimates of the VSL [17], each of which yields an average across-country income elasticity estimate of 1.0 using a procedure that accounts for publication selection effects. These calculations use the same income elasticity for all countries but estimate country-specific levels of VSL taking into account the income level in each country as compared to that in the U.S. Drawing on these estimates and other studies, the U.S. Environmental Protection Agency [18] also adopted an income elasticity of 1.0 to transfer U.S. VSL estimates to countries that do not have VSL estimates in their work estimating the global mortality cost component of the social cost of carbon.

Countries with income levels much lower than the U.S. tend to have VSL levels that are more responsive to their lower income levels, making it desirable to explore the sensitivity of the results to different assumptions about the income elasticity of the VSL for low- and middle-income countries. Column 2 of Table A1 in S1 Appendix presents the baseline estimates using an income elasticity of 1.0, listing the VSL for countries throughout the world calculated based on this estimate and the associated COVID-19 mortality costs. Section 3.3 below presents results using other income elasticities that can be used to transfer the U.S. VSL to construct country-specific values, with the overall effect being that higher (lower) income elasticities tend to reduce (raise) the estimates of the global mortality costs for most countries given the relatively high U.S. GNI per capita. To the extent that countries with lower income levels than the U.S. have income elasticities greater than 1.0, the mortality cost estimates for COVID-19 will be reduced for these countries.

By the end of 2022, the total global mortality cost was $29.4 trillion, which is a per capita cost of $3,818. The cost levels by year were $9.0 trillion in the 2019 to 2020 period, $13.1 trillion in 2021, and $7.3 trillion in 2022. Table 1 summarizes these values by year and for the top ten mortality cost countries in each of the time periods indicated in the five panels of Table 1.

The U.S. was the largest contributor to the global mortality costs, with total costs of $12.7 trillion, or per capita costs of $38,370, which are an order of magnitude greater than the worldwide average. The costs by year were $4.1 trillion through 2020, $5.6 trillion in 2021, and $3.1 trillion in 2022. These figures do not imply that the government would have spent these amounts on a single policy that would have eliminated the risks. Since the available policy choices generally pertain to minor reductions in mortality risks rather than complete elimination of COVID risks, concerns regarding whether an expenditure of this magnitude would have been feasible do not arise.

Notwithstanding the considerable temporal variation in the U.S. mortality costs, the relative role of U.S. mortality costs has been remarkably invariant for all five panels of the time periods shown in Table 1. Although the U.S. has 4% of the global population, the U.S. share of global mortality costs was 45% through 2020 (Panel A), 43% in 2021 (Panel B), 44% through 2021 (Panel C), 43% in 2022 (Panel D), and 43% through 2022. The U.S. fluctuations in mortality costs consequently track the worldwide mortality cost pattern quite closely.

Several other affluent countries have consistent prominent roles in the worldwide COVID mortality costs, in part because of their relatively high VSL. The United Kingdom has an average VSL of $8.8 million, which contributed to it having the second highest mortality cost at the end of 2020, the fourth highest mortality cost in 2021, and the third highest mortality costs in 2022, leading to it being the second ranking country in terms of total mortality costs through 2022. Germany has a VSL of $9.4 million and had the third highest in mortality costs through 2022 as a result of having the sixth highest cost through 2020, the third highest cost in 2021, and the second highest cost in 2022.

Despite having lower income levels and a lower VSL, Brazil has had a prominent ranking in terms of total mortality costs. Brazil is also noteworthy in that the large number of deaths in Brazil offsets the impact of a lower VSL of $2.0 million. Brazil ranked fifth in total mortality costs at the end of 2020, was second in 2021, and did not make the top 10 in 2022 despite 74,832 COVID deaths, which is more than the number of deaths for all but two of the countries listed in the top 10 in Panel D of Table 1. The combined influence of the relatively low VSL and large number of deaths resulted in Brazil finishing fourth overall for the cumulative mortality costs at the end of 2022.

### 3.3 Alternative assumptions regarding the income elasticity of the VSL

The extrapolation of the U.S. VSL to other countries thus far was based on the assumption that the country-specific VSL could be constructed using an income elasticity of 1.0. Other assumptions that can be used to transfer the U.S. VSL to other countries are also possible. For example, if all countries were accorded the same VSL as the U.S., the total global mortality cost of the pandemic would be $75.8 trillion rather than $29.4 trillion. The ordering and relative values of the countries’ mortality costs would be the same as the number of COVID-related deaths. One might suggest that applying a uniform VSL for all countries is the correct approach on the grounds that all lives are equally valuable. However, the mortality cost estimates do not set out to measure the intrinsic moral value of these lives. Rather, the mortality cost calculations measure society’s willingness to pay to reduce mortality risks in these different countries. This valuation of risks varies with the income levels in those countries just as do most other categories of expenditures. Moreover, it is not likely that a country’s VSL that emerges based on an income elasticity of 1.0 generally understates the country-specific VSL since the VSL based on this income elasticity is greater than the VSL that government agencies in these countries use for policy analyses [12].

Use of an elasticity of 1.0 is consistent with U.S. Department of Transportation [19] practices for modifying the U.S. VSL in response to changes in income, but there is more diversity in the VSL income elasticity assumptions used in analyses outside the U.S. A prominent alternative economic approach is to suggest that the VSL for low- and middle-income countries outside the U.S. should be based on an income elasticity greater than 1.0 [20–23]. Adopting a formulation that leads to a more than proportional relationship of the VSL and income will lead to an even lower VSL for these lower income countries than would prevail with an elasticity of 1.0. The rationales that Hammitt and Robinson [20] and Robinson, Hammitt, and O’Keefe [23] offer for using a higher elasticity estimate for lower income countries are based on across-country comparisons of the VSL and the high percentage of income that would be devoted to risk reduction based on the use of a unitary income elasticity. Robinson, Hammitt, and O’Keefe [23] propose that policy analyses include a sensitivity analysis based on an income elasticity of 1.5 for low- and middle-income countries, which will be done below. Hoffman, Krupnick, and Qin [24] have also questioned the common use of a unitary income elasticity for international VSL estimates, but found a different relationship. Their study drew on a set of studies fielding the same stated preference VSL valuation instrument in Canada, China, France, Italy, Japan, Mongolia, the United Kingdom, and the U.S. They found estimated VSL-transfer income elasticities slightly below 1.0 for lower-income countries, implying a smaller diminution of the VSL for low-income countries than would be predicted based on an income elasticity of 1.0. The World Bank study by Narain and Sall [21] adopted an income elasticity of 1.2 for transferring the VSL to low- and middle-income countries. The World Bank and the Institute for Health Metrics and Evaluation [22] analysis of international air pollution costs adopted an elasticity of 1.2 for low- and middle-income countries and 0.8 for high-income countries. OECD [25] recommended an elasticity estimate of 0.8 for OECD countries. The OECD [26] assessment of international mortality costs of air pollution adopted greater income elasticities for low- and middle-income countries but started at a lower elasticity base level for high-income countries. That OECD [26] report used income elasticities of 0.8 for high-income countries, 0.9 for middle-income countries, and 1.0 for low-income countries.

Although the average estimated international income elasticity of the VSL of 1.0 is consistent with the U.S. Environmental Protection Agency’s [18] use of an international income elasticity of 1.0, many empirical estimates of the income elasticity of the VSL are greater for low- and middle-income countries. In the international VSL meta-regression analysis by Viscusi and Masterman [16], the estimated income elasticity at the 10^th^ percentile of the international VSL distribution was 1.5, which would imply a 1.5% drop in the VSL if a country has a 1% lower income level than the U.S. The income elasticity at the 25^th^ percentile of the VSL distribution across different countries was 1.2, which would entail a reduction of the VSL by 1.2% for a 1% lower income level. Applying these higher income elasticities has little effect on high-income countries but has a greater impact on the VSL for lower income countries. The VSL for the United Kingdom is $8.8 million based on an elasticity of 1.0, $8.4 million using an income elasticity of 1.2, and $7.8 million using an income elasticity of 1.5. The VSL for Thailand drops more steeply from a VSL of $1.2 million at an income elasticity of 1.0, to $0.7 million with an income elasticity of 1.2, and to $0.4 million with an income elasticity of 1.5. A very low-income country such as Bangladesh has a VSL of $240,000 for an elasticity of 1.0, $110,000 for an elasticity of 1.2, and $40,000 for an elasticity of 1.5.

It is instructive to explore the implications of different income elasticity assumptions of 1.0, 1.2, and 1.5. In addition to presenting estimates transferring the U.S. VSL to other countries based on an income elasticity of 1.0, Table 2 reports results using an upper bound elasticity of 1.5 and an income elasticity of 1.2, both of which have appeared in previous empirical studies of the VSL as well as in policy analyses. The calculation of the *VSL*_*i*_ for any country *i* using information on the *VSL*_*US*_ in the U.S., the income *Y*_*i*_ in the country *i*, the income *Y*_*US*_ in the U.S., and the income elasticity, *ε*, is given by

$${VSL}_{i}={VSL}_{US}*{\left({Income}_{i}/{Income}_{US}\right)}^{\varepsilon }.$$

**Table 2:** Cumulative COVID-19 mortality costs with different income elasticities.

**Panel A** Cumulative COVID-19 Mortality Costs (Millions $) as of January 1, 2021				
		**Income Elasticity**		
		**1**	**1.2**	**1.5**
**GNI per Capita Percentile**	**76–100**	7,398,983	7,195,776	6,930,240
	**51–75**	1,436,539	1,013,884	603,524
	**26–50**	87,231	50,294	22,101
	**0–25**	56,757	27,644	9,410
	TOTAL	8,979,510	8,287,598	7,565,275
**Panel B** Cumulative COVID-19 Mortality Costs (Millions $) as of January 1, 2022				
		**Income Elasticity**		
		**1**	**1.2**	**1.5**
**GNI per Capita Percentile**	**76–100**	16,716,295	16,271,600	15,692,899
	**51–75**	4,757,640	3,364,355	2,008,500
	**26–50**	429,899	248,564	109,793
	**0–25**	189,284	91,928	31,176
	**TOTAL**	22,093,118	19,976,447	17,842,368
**Panel C** Cumulative COVID-19 Mortality Costs (Millions $) as of January 1, 2023				
		**Income Elasticity**		
		**1**	**1.2**	**1.5**
**GNI per Capita Percentile**	**76–100**	23,088,188	22,453,193	21,626,523
	**51–75**	5,548,852	3,932,435	2,355,279
	**26–50**	512,118	296,741	131,548
	**0–25**	207,313	100,715	34,171
	**TOTAL**	29,356,470	26,783,085	24,147,520

The mortality cost statistics in Table 2 are shown separately for different groups of countries based on their per capita GNI levels. The bottom income quartile extends from Burundi to Sao Tome and Principe. The 26^th^–50^th^ quartile includes countries from Sudan and the Solomon Islands to Thailand. The 51^st^–75^th^ quartile has a per capita GNI range from Iraq to Oman. Finally, the countries in the top quartile per capita GNI range extend from Slovakia to Bermuda. Panel A pertains to the total costs through 2020, Panel B summarizes the total costs through 2021, and Panel C summarizes the total costs through 2022.

Since the pattern of mortality cost values is similar across all panels, let us focus on the most recent cumulative results in Panel C. The extent to which the mortality costs are affected by use of different income elasticities varies by quartile in the expected fashion. The top quartile is least affected, as there is only a 6% drop in the mortality costs after switching the income elasticity from 1.0 to 1.5. The bottom quartile estimates change much more substantially, exhibiting an 84% decline as a result of the elasticity increase from 1.0 to 1.5. However, because the value of the total global mortality costs is driven principally by the value of fatalities in the upper income countries, the value of the global mortality costs only declines by 18% if one adopted an elasticity of 1.5 throughout the calculations rather than 1.0. In that case, the U.S. mortality cost share of global mortality costs is 53%. The intermediate income elasticity of 1.2 leads to a 9% decline in global mortality costs. The U.S. share of the global mortality costs is 48% in this intermediate case.

Using the different results in Table 2, it is also possible to fine tune the mortality cost estimates if one wishes to assign different income elasticities to different income groups. For example, suppose that the mortality costs through January 1, 2023, have an income elasticity of 1.0 for the top two quartiles, 1.2 for the 26^th^–50^th^ quartile, and 1.5 for the 0–25^th^ quartile. Then the total global mortality cost is $29.0 trillion which is 1% less than cumulative mortality cost shown in Panel E of Table 1. If instead one had applied an income elasticity of 1.5 for the 0–25^th^ quartile and 1.2 throughout the 26–75^th^ quartiles, and 1.0 for the highest quartile, the total mortality cost would have been $27.4 trillion which is a 7% reduction from the income elasticity of 1.0 case.

## 4. The U.S. outlier status

Whether the U.S. is an outlier in terms of its mortality record depends in part on what influences are recognized. To provide a summary assessment, Table 3 presents regression results that indicate the correlations with the dependent variable, the log of the COVID mortality rate. Death rates are calculated using COVID death and population data reported by Worldometer [1] on January 1, 2021, 2022, and 2023. VSL data is calculated as described in section 3.2 above—an elasticity of 1.0 is used for calculating the VSL estimates. Population age data is derived from United Nations [27] estimates by five-year age group in 2021. Urban population data is also retrieved from Worldometer [28] data as of March 11, 2020 (the day the WHO declared the pandemic). Healthcare quality rankings are from a 2000 study commissioned by the WHO [29]. Column 1 pertains to the cumulative death rate through January 1, 2023, column 2 presents the results for 2022, and column 3 presents the results for 2021.

**Table 3:** Log COVID-19 death rate regressions.

	(1)	(2)	(3)
VARIABLES	Log Total Death Rate through January 1, 2023	Log Death Rate in 2022	Log Death Rate in 2021
Log of the VSL	0.770***	1.028***	0.660***
	(0.125)	(0.151)	(0.178)
United States Indicator	0.389	0.183	0.762
	(1.252)	(1.498)	(1.772)
Log Percent of Population over 65	-0.0237	0.232	0.180
	(0.322)	(0.387)	(0.457)
Log Percent of Urban Population	0.320	-0.222	0.185
	(0.280)	(0.340)	(0.414)
Health Rank Percentile 76–100	-0.163	0.651	-0.359
	(0.455)	(0.556)	(0.647)
Health Rank Percentile 51–75	0.927***	1.430***	1.165**
	(0.335)	(0.406)	(0.480)
Health Rank Percentile 26–50	0.263	0.757**	0.119
	(0.301)	(0.369)	(0.433)
Constant	4.525***	5.089***	5.044**
	(1.451)	(1.761)	(2.128)
Log of the VSL			
Observations	168	167	165
R-squared	0.527	0.573	0.302

Notes: Standard errors in parentheses

*** p<0.01

** p<0.05

* p<0.1

The principal variable of interest is the log VSL, which relates the mortality rate to each country’s valuation of mortality risks as indicated by the VSL. Because the VSL variable is the income-adjusted U.S. VSL amount, the variable in effect is a proxy for across-country differences in income levels. To the extent that higher income levels and the associated higher VSL level boost the valuation of health risks, one would have expected that the VSL would be negatively associated with the mortality rates. However, the opposite pattern is observed. The log VSL variable has a coefficient of 0.77, indicating that a 10% increase in the VSL will be associated with a 7.7% increase in the COVID mortality rate. The estimates for 2022 imply a proportional relationship, while the estimates for 2021 indicate that the VSL rises less than proportionally with the VSL. More affluent countries overall tended to experience higher mortality rates, which is inconsistent with their greater valuation of mortality costs. This result may be due in part to the structure of more advanced economies or their reluctance to undertake preventive actions because of the greater economic opportunity costs involved. An alternative hypothesis is that more affluent countries are more vigilant in identifying and reporting COVID-related fatalities, so that the results reflect a reporting bias rather than differences in mortality rates.

After controlling for the log VSL and other demographic factors in Table 3, the categorical variable for the U.S. is positive but not statistically significant. There is no apparent additional U.S. influence other than that through the VSL. Put somewhat differently, the U.S. performance is not an outlier after controlling for the VSL and the other variables in the analysis.

The only other variable that is statistically significant pertains to the overall ranking of the health care systems in different countries, where these are distinguished by their quartile rank. Compared to the 0–25^th^ health care quartile, there are higher fatality rates for countries in the 51^st^ to 75^th^ quartile, and in the case of the 2022 results, for the 26^th^ to 50^th^ quartile as well. These correlations may reflect more vigilant monitoring of COVID-related illnesses compared to the least affluent countries. The absence of a statistically significant influence of the population over 65 is surprising.

## 5. Implications and limitations

Monetizing the mortality costs of COVID-19 consists of two components, the number of fatalities and the value attached to these fatalities based on the VSL. Because the U.S. is a leader in both dimensions, examining the impact of the pandemic in terms of mortality costs enhances the outlier status of the U.S. While the U.S. leads the world in total COVID-related mortality, the relative gap between the U.S. and the rest of the world in terms of monetized COVID-19 costs is even greater than the disparity in COVID-related deaths because of the relatively high VSL level in the U.S. Whereas the U.S. accounted for 17% of global COVID-related deaths through 2021, it accounted for 43% of the global mortality costs. In percentage terms, the U.S. share of global mortality costs is more than double its share of global deaths. This percentage share has been relatively invariant, ranging from 43% to 45% throughout the pandemic. The global mortality costs through 2022 is $29.4 trillion. Given the substantial mortality cost weight that the U.S. and other affluent countries receive in the calculations, the estimates are not greatly affected by reasonable alternative assumptions regarding how the costs for lower income countries are calculated.

The global focus of these estimates of the COVID-related mortality costs entailed a variety of assumptions needed to make the analysis feasible. The number of COVID-related deaths is based on Worldometer data, which are consistent with the WHO estimates. In each case, the principal source of data consists of reports by governmental health organizations in the different countries, but these entities may differ in the vigilance of their reporting of COVID-related deaths. Consideration of health impacts was restricted to fatalities so that additional losses for those who do not die but nevertheless suffer COVID-related illnesses were not included.

The monetization of the deaths using the VSL embodied several assumptions. Application of the VSL to COVID-related deaths in effect treats the valuation of the risk of these illness-related deaths as being comparable to that for the traumatic deaths that constitute the majority of occupational fatalities. If the morbidity losses associated with COVID-related fatalities are greater than for acute injuries, application of the standard VSL estimates would understate the mortality cost. A countervailing influence is that if the VSL is lower for the older population most susceptible to COVID-related mortality, the shorter lifespans at risk would reduce the monetized mortality cost by about half if the international age distribution of deaths and the VSL are comparable to those values for the U.S. Extrapolating the U.S. VSL to other countries was based on the assumption that the average VSL for different countries varied principally in response to differences in per capita income levels. Differences in the income-VSL relationship did not have a great impact on the estimated mortality costs, but there could be other differences in risk preferences by country that are influential as well.

Application of a broad range of assumptions regarding the income-VSL relationship does not greatly alter the results. The main reason that the mortality cost estimates are robust with respect to different valuations is that the largest number of COVID-related fatalities occur in the U.S., which also has the highest VSL. Other high-income countries also have high mortality rates and high VSL levels that are not too far below US levels. Although the scope of the analysis is global, the distribution of the monetized value of the COVID-related illnesses is concentrated among the U.S. and other high-income countries.

## Supporting information

S1 Appendix (DOCX)
