# Supplementary material for: The global COVID-19 mortality cost report card: 2020, 2021, and 2022
Source: PLoS One. 2023 May 11;18(5):e0284273. doi: 10.1371/journal.pone.0284273 (PMC10174491; doi:10.1371/journal.pone.0284273)
Supplement: S1 Appendix — (DOCX) [file pone.0284273.s001.docx]

**Appendix**

| **Table A1: The Global Mortality Costs of COVID-19 as of January 1, 2023** | | | |  |
| --- | --- | --- | --- | --- |
|  |  |  |  |  |
| **Country** | **Number of Deaths** | **VSL with Elasticity of 1.0 (Millions $)** | **VSL x Deaths (Millions $)** |  |
| World | 6,649,477 | - | 29,356,470 |  |
| Afghanistan | 7,849 | 0.12 | 975 |  |
| Albania | 3,595 | 0.87 | 3,133 |  |
| Algeria | 6,881 | 0.99 | 6,824 |  |
| Andorra | 165 | 8.81 | 1,454 |  |
| Angola | 1,930 | 0.85 | 1,643 |  |
| Antigua and Barbuda | 146 | 2.70 | 395 |  |
| Argentina | 130,124 | 2.54 | 330,177 |  |
| Armenia | 8,716 | 0.79 | 6,887 |  |
| Australia | 17,052 | 12.23 | 208,595 |  |
| Austria | 21,443 | 9.65 | 207,027 |  |
| Azerbaijan | 10,008 | 1.34 | 13,370 |  |
| Bahamas | 833 | 4.22 | 3,518 |  |
| Bahrain | 1,539 | 4.04 | 6,218 |  |
| Bangladesh | 29,440 | 0.24 | 7,134 |  |
| Barbados | 568 | 2.95 | 1,678 |  |
| Belarus | 7,118 | 1.32 | 9,364 |  |
| Belgium | 33,228 | 9.01 | 299,426 |  |
| Belize | 688 | 0.91 | 629 |  |
| Benin | 163 | 0.17 | 28 |  |
| Bermuda | 153 | 21.61 | 3,307 |  |
| Bhutan | 21 | 0.48 | 10 |  |
| Bolivia | 22,299 | 0.61 | 13,623 |  |
| Bosnia and Herzegovina | 16,227 | 0.95 | 15,432 |  |
| Botswana | 2,794 | 1.32 | 3,676 |  |
| Brazil | 693,941 | 2.01 | 1,391,973 |  |
| Brunei | 225 | 7.74 | 1,742 |  |
| Bulgaria | 38,108 | 1.52 | 58,048 |  |
| Burkina Faso | 395 | 0.13 | 51 |  |
| Burundi | 38 | 0.05 | 2 |  |
| Cabo Verde | 412 | 0.67 | 275 |  |
| Cambodia | 3,056 | 0.22 | 666 |  |
| Cameroon | 1,965 | 0.27 | 528 |  |
| Canada | 48,948 | 9.68 | 473,877 |  |
| Central African Republic | 113 | 0.07 | 8 |  |
| Chad | 194 | 0.18 | 35 |  |
| Chile | 63,200 | 2.87 | 181,471 |  |
| China | 5,249 | 1.61 | 8,477 |  |
| Colombia | 142,179 | 1.45 | 206,731 |  |
| Comoros | 161 | 0.16 | 26 |  |
| Congo, Dem. Rep. | 1,462 | 0.08 | 122 |  |
| Congo, Rep. | 386 | 0.52 | 200 |  |
| Costa Rica | 9,072 | 2.12 | 19,214 |  |
| Croatia | 17,607 | 2.59 | 45,537 |  |
| Cyprus | 1,258 | 5.29 | 6,658 |  |
| Czech Republic (Czechia) | 42,134 | 3.69 | 155,647 |  |
| Denmark | 7,747 | 11.92 | 92,370 |  |
| Dominica | 74 | 1.38 | 102 |  |
| Dominican Republic | 4,384 | 1.27 | 5,571 |  |
| Ecuador | 35,940 | 1.23 | 44,133 |  |
| Egypt | 24,613 | 0.68 | 16,741 |  |
| El Salvador | 4,230 | 0.80 | 3,394 |  |
| Equatorial Guinea | 183 | 2.61 | 478 |  |
| Estonia | 2,854 | 3.74 | 10,671 |  |
| Ethiopia | 7,572 | 0.12 | 910 |  |
| Fiji | 881 | 0.98 | 867 |  |
| Finland | 7,933 | 9.48 | 75,202 |  |
| France | 161,962 | 8.26 | 1,337,115 |  |
| Gabon | 306 | 1.87 | 573 |  |
| Gambia | 372 | 0.09 | 35 |  |
| Georgia | 16,897 | 0.85 | 14,314 |  |
| Germany | 161,465 | 9.36 | 1,510,572 |  |
| Ghana | 1,461 | 0.30 | 440 |  |
| Greece | 34,779 | 4.14 | 143,917 |  |
| Grenada | 238 | 1.76 | 419 |  |
| Guatemala | 20,001 | 0.73 | 14,622 |  |
| Guinea | 466 | 0.10 | 45 |  |
| Guinea-Bissau | 176 | 0.12 | 21 |  |
| Guyana | 1,286 | 0.83 | 1,071 |  |
| Haiti | 860 | 0.16 | 142 |  |
| Honduras | 11,071 | 0.46 | 5,140 |  |
| Hong Kong | 11,869 | 8.35 | 99,099 |  |
| Hungary | 48,495 | 2.64 | 128,187 |  |
| Iceland | 229 | 10.21 | 2,338 |  |
| India | 530,705 | 0.33 | 172,920 |  |
| Indonesia | 160,619 | 0.70 | 112,519 |  |
| Iran | 144,688 | 1.33 | 192,995 |  |
| Iraq | 25,373 | 1.19 | 30,072 |  |
| Ireland | 8,293 | 10.71 | 88,798 |  |
| Isle of Man | 116 | 17.37 | 2,015 |  |
| Israel | 12,037 | 7.28 | 87,682 |  |
| Italy | 184,642 | 6.68 | 1,233,697 |  |
| Jamaica | 3,461 | 1.03 | 3,559 |  |
| Japan | 57,513 | 7.91 | 454,901 |  |
| Jordan | 14,122 | 0.95 | 13,459 |  |
| Kazakhstan | 13,695 | 2.32 | 31,766 |  |
| Kenya | 5,688 | 0.27 | 1,552 |  |
| Korea, Rep. (South Korea) | 32,219 | 5.59 | 180,105 |  |
| Kuwait | 2,570 | 8.58 | 22,060 |  |
| Kyrgyzstan (Kyrgyz Republic) | 2,991 | 0.24 | 713 |  |
| Laos (Lao People's Dem. Rep.) | 758 | 0.35 | 269 |  |
| Latvia | 6,161 | 3.05 | 18,795 |  |
| Lebanon | 10,746 | 1.57 | 16,872 |  |
| Lesotho | 706 | 0.26 | 184 |  |
| Liberia | 294 | 0.08 | 23 |  |
| Lithuania | 9,485 | 3.04 | 28,858 |  |
| Luxembourg | 1,133 | 15.68 | 17,766 |  |
| Macao | 47 | 13.68 | 643 |  |
| Macedonia (North Macedonia) | 9,614 | 1.05 | 10,063 |  |
| Madagascar | 1,416 | 0.09 | 121 |  |
| Malawi | 2,685 | 0.07 | 186 |  |
| Malaysia | 36,857 | 2.15 | 79,335 |  |
| Maldives | 311 | 1.42 | 440 |  |
| Mali | 743 | 0.15 | 115 |  |
| Malta | 817 | 4.87 | 3,981 |  |
| Marshall Islands | 17 | 0.97 | 17 |  |
| Mauritania | 997 | 0.28 | 278 |  |
| Mauritius | 1,041 | 1.99 | 2,073 |  |
| Mexico | 331,099 | 1.98 | 654,710 |  |
| Moldova | 11,933 | 0.46 | 5,443 |  |
| Mongolia | 2,179 | 0.79 | 1,717 |  |
| Montenegro | 2,791 | 1.47 | 4,104 |  |
| Morocco | 16,295 | 0.62 | 10,055 |  |
| Mozambique | 2,230 | 0.12 | 268 |  |
| Myanmar | 19,490 | 0.24 | 4,604 |  |
| Namibia | 4,082 | 1.06 | 4,314 |  |
| Nepal | 12,019 | 0.15 | 1,787 |  |
| Netherlands | 22,989 | 9.95 | 228,742 |  |
| New Zealand | 3,513 | 8.15 | 28,630 |  |
| Nicaragua | 225 | 0.40 | 89 |  |
| Niger | 312 | 0.08 | 25 |  |
| Nigeria | 3,155 | 0.57 | 1,812 |  |
| Norway | 4,571 | 19.09 | 87,259 |  |
| Oman | 4,260 | 3.44 | 14,670 |  |
| Pakistan | 30,636 | 0.29 | 8,984 |  |
| Panama | 8,567 | 2.42 | 20,726 |  |
| Papua New Guinea | 669 | 0.46 | 305 |  |
| Paraguay | 19,688 | 0.85 | 16,799 |  |
| Peru | 218,232 | 1.25 | 272,427 |  |
| Philippines | 65,397 | 0.72 | 47,278 |  |
| Poland | 118,533 | 2.72 | 322,008 |  |
| Portugal | 25,714 | 4.18 | 107,505 |  |
| Qatar | 685 | 17.10 | 11,716 |  |
| Romania | 67,374 | 1.93 | 130,343 |  |
| Russia | 393,762 | 2.33 | 918,145 |  |
| Rwanda | 1,467 | 0.14 | 209 |  |
| Samoa | 29 | 0.80 | 23 |  |
| Sao Tome and Principe | 77 | 0.36 | 28 |  |
| Saudi Arabia | 9,520 | 4.80 | 45,656 |  |
| Senegal | 1,968 | 0.20 | 393 |  |
| Serbia | 17,526 | 1.13 | 19,773 |  |
| Seychelles | 172 | 3.01 | 517 |  |
| Sierra Leone | 126 | 0.13 | 16 |  |
| Singapore | 1,711 | 10.61 | 18,150 |  |
| Slovakia | 20,823 | 3.58 | 74,505 |  |
| Slovenia | 7,008 | 4.52 | 31,668 |  |
| Solomon Islands | 153 | 0.39 | 60 |  |
| South Africa | 102,568 | 1.24 | 126,995 |  |
| South Sudan | 138 | 0.16 | 22 |  |
| Spain | 117,095 | 5.81 | 680,318 |  |
| Sri Lanka | 16,817 | 0.77 | 13,014 |  |
| St. Kitts and Nevis | 46 | 3.07 | 141 |  |
| St. Lucia | 409 | 1.50 | 612 |  |
| St. Vincent (St. Vincent and the Grenadines) | 119 | 1.35 | 161 |  |
| Sudan | 4,994 | 0.39 | 1,953 |  |
| Suriname | 1,393 | 1.91 | 2,655 |  |
| Swaziland (Eswatini) | 1,422 | 0.67 | 950 |  |
| Sweden | 21,795 | 11.80 | 257,074 |  |
| Switzerland | 14,371 | 17.23 | 247,676 |  |
| Tajikistan | 125 | 0.26 | 33 |  |
| Tanzania | 845 | 0.19 | 158 |  |
| Thailand | 33,594 | 1.16 | 39,132 |  |
| Timor-Leste | 138 | 0.44 | 61 |  |
| Togo | 290 | 0.11 | 32 |  |
| Trinidad and Tobago | 4,283 | 3.59 | 15,386 |  |
| Tunisia | 29,284 | 0.81 | 23,735 |  |
| Turkey | 101,492 | 2.03 | 205,649 |  |
| Uganda | 3,630 | 0.14 | 517 |  |
| Ukraine | 110,766 | 0.54 | 59,550 |  |
| United Arab Emirates | 2,348 | 8.78 | 20,604 |  |
| United Kingdom | 198,937 | 8.84 | 1,757,831 |  |
| United States | 1,117,988 | 11.40 | 12,745,063 |  |
| Uruguay | 7,562 | 3.20 | 24,208 |  |
| Uzbekistan | 1,637 | 0.44 | 720 |  |
| Vanuatu | 14 | 0.65 | 9 |  |
| Vietnam | 43,186 | 0.41 | 17,501 |  |
| West Bank and Gaza (Palestine) | 5,404 | 0.63 | 3,401 |  |
| Yemen | 2,159 | 0.23 | 501 |  |
| Zambia | 4,024 | 0.30 | 1,221 |  |
| Zimbabwe | 5,637 | 0.18 | 987 |  |

Notes: Data on COVID deaths are pulled from Worldometer as reported on January 1, 2023, which were retrieved using the Wayback Machine digital internet archive. The 23:12:53 snapshot was used and is available here: https://web.archive.org/web/20230101231253/https:/www.worldometers.info/coronavirus/. The VSL for various countries was calculated using the methodology described by Viscusi and Masterman [16] where the calculation of the *VSL_i_* for any country *i* using information on the *VSL_US_* in the U.S., the income *Y_i_* in the country *i,* the income *Y_US_* in the U.S., and the income elasticity, *ε*, is given by ${VSL}_{i}= {VSL}_{US}*\left( {{Income}_{i}}/{{Income}_{US}} \right)^{\varepsilon}$. An income elasticity of 1.0 and a base U.S. VSL of 11.4 million USD adjusted for inflation to December 2021 dollars are utilized. The base U.S. VSL is derived from a meta-analysis of VSL studies using labor market data to assess the wage increase that workers require to incur fatality risks [11]. Gross national income (GNI) per capita data reported by Viscusi and Masterman [16] are used as measures of the income in each county, including the U.S. While Viscusi and Masterman [16] reported GNI data for 189 countries, Worldometer did not report COVID death data for all of these countries in 2021, 2022, and 2023. Countries that did not appear in the Worldometer data for all three observation periods were excluded even though Viscusi and Masterman [16] provide GNI data. These dropped countries were Cote d’Ivoire, Kiribati, Kosovo, Micronesia, Nauru, Palau, Puerto Rico, Tonga, Turkmenistan, and Tuvalu.
